# Measuring the effectiveness of hybrid diabetes care over 90 days through continuous data monitoring in type 2 diabetic patients

**DOI:** 10.3389/fendo.2024.1355792

**Published:** 2024-05-07

**Authors:** Hala Zakaria, Yousef Said, Sofia Aleabova, Jestoni Bangayan, Mirabelle Dandan, Joelle Debs, Nichole Dahlstrom, Dianne Divino, Ali Hashemi, Ihsan Almarzooqi

**Affiliations:** GluCare Integrated Diabetes Center, Dubai, United Arab Emirates

**Keywords:** emote continuous data monitoring, diabetes, continuous glucose monitoring, engagement, hybrid healthcare, data-driven personalized medicine, virtual monitoring, HbA1c reduction

## Abstract

**Background:**

Diabetes Mellitus, a global health challenge, affects 537 million individuals. Traditional management relies on periodic clinic visits, but technological advancements, including remote monitoring, offer transformative changes. Telemedicine enhances access, convenience, adherence, and glycemic control. Challenges include trust-building and limitations in face-to-face interactions. Integrating remote monitoring with in-person healthcare creates a hybrid approach. This study evaluates the impact on Type 2 Diabetes patients over 3 months.

**Methods:**

A retrospective case-control observational study. Inclusion criteria involved previous Type 2 Diabetes diagnosis and a minimum 3-month GluCare model period with two physical visits. Patients in the case group had in-clinic visits, bi-weekly app engagement, and monthly body weight readings. Control group had in-clinic visits only. Outcomes measured included HbA1c, lipid profile, CV risk, eGFR, urine Albumin/Creatinine Ratio, Uric Acid, and CRP.

**Results:**

Case group showed significant HbA1c improvements (-2.19%), especially in higher baseline levels. Weight, BMI, LDL, total cholesterol, and CVD risk also improved. Controls showed smaller improvements. Higher digital interactions correlated with better outcomes. Patients with ≥11 interactions showed significant reductions in HbA1c (-2.38%) and weight (-6.00 kg).

**Conclusion:**

The GluCare.Health hybrid model demonstrates promising outcomes in Type 2 diabetes management. The integration of in-clinic consultations with continuous remote monitoring leads to substantial improvements in glycemic control and clinical parameters. The study highlights the importance of patient engagement in achieving positive outcomes, with higher digital interactions associated with greater reductions in HbA1c and weight. The hybrid approach proves more effective than digital-only interventions, emphasizing the need for comprehensive, end-to-end solutions in diabetes care.

## 1 Introduction

Diabetes Mellitus, a chronic metabolic disorder, presents a formidable challenge to healthcare systems worldwide. The global prevalence of diabetes has reached staggering proportions, with 537 million individuals affected, and projections indicating a relentless upward trend (1). In the UAE, the International Diabetes Federation (IDF) reported the prevalence of diabetes at 16.3%, which is significantly higher than the global average of 9.3% (2). By 2045, it is predicted that 23.4% of the population between 20–79 years old in the UAE will have diabetes (3). Therefore, the local impact of diabetes reveals a complex web of challenges that include not just health implications but also significant economic burdens. At the local level, diabetes management encounters unique hurdles, such as variations in healthcare accessibility, cultural dietary habits, and lifestyle choices that exacerbate the prevalence and management of the disease (4, 5).

The intricate nature of diabetes management and the potential for severe complications necessitate innovative approaches to enhance patient care and quality of life. Traditionally, diabetes management relied on periodic clinic visits and individual self-monitoring efforts. However, the rapid strides in technology have ushered in a new era of healthcare, promising transformative changes in the management of this chronic condition. The emergence of continuous remote monitoring, facilitated by wearable devices and digital health platforms, offers a paradigm shift in patient-provider interaction (6). This technological advancement empowers real-time data collection, initially through monitoring of glucose level fluctuations, and the potential for timely interventions, thereby redefining the contours of diabetes care (6). New digital biomarkers such as sleep data and heart rate are also starting to be used in digital health and can help drive behavioral changes that are much needed in overall diabetes management.

The utilization of telemedicine in diabetes care offers several advantages. It enhances access to healthcare providers, contributing to improved healthcare accessibility (6), patient convenience, increased adherence to scheduled medical appointments, and the enhancement of glycemic control (7). Furthermore, it holds the potential to reduce administrative healthcare costs, rendering it an economically efficient approach (8). This heightened accessibility has the potential to enhance patient engagement and glycemic control, however, digital health in diabetes care faces various limitations. Firstly, there is the critical issue of trust-building that comes from face-to-face interactions, as patients must feel at ease and trust the quality of care if they receive any care remotely (6). Moreover, the inherent limitation of telemedicine, which restricts face-to-face interaction, is particularly challenging as some aspects of diabetes education, behavioral therapy, and counseling may necessitate in-person engagement, potentially impacting the depth and quality of the patient-provider relationship (6). Finally, digital health tools need to be an intrinsic part of the care model offered by providers, rather than an adjunct tool offered separately. Many Digital-only offerings do not have leverage on important decision making ability such as prescription behavior or access to Electronic Medical Records (EMR) (9).

Hybrid care models, integrating remote data monitoring with physical healthcare creates a synergistic approach that seeks to overcome these challenges. By combining the strengths of both modalities, healthcare providers can establish a more comprehensive, hybrid and integrated approach (9–11). Remote monitoring can be utilized for regular check-ins, continuous monitoring of vital health metrics, remote medication management, and facilitating virtual consultations with a greater team beyond just physicians. Meanwhile, in-person interactions can address trust-building issues and cater to aspects of care that benefit from face-to-face engagement, such as hands-on examinations, more personal counseling, diagnostic modalities and immediate feedback (12). It is well documented that engagement, regardless of delivery mode, is correlated to improved outcomes within diabetes management (13–16).

The GluCare.Health Model, implemented by GluCare a diabetes center in Dubai, UAE, represents an a hybrid approach to diabetes management, integrating both in-clinic and digital components to encourage behavioral change and effective disease management. The in-clinic component adheres to the American Diabetes Association’s (ADA) standards, offering personalized care through a dedicated team of healthcare professionals. This team includes physicians, dietitians, diabetes nurse educators, and health coaches. Complementing the in-clinic visits, the Remote Continuous Data Monitoring (RCDM) system provides a digital means of continuous care, allowing for real-time tracking and analysis of diabetes-related metrics. This includes glucose levels through Continuous Glucose Monitoring (CGM), sleep quality, dietary intake, physical activity, and body weight. The RCDM enables remote medication adjustments, personalized dietary feedback via a mobile app, and direct engagement with healthcare professionals for medication management. Moreover, the program places a strong emphasis on education, utilizing a QISMET-accredited curriculum to deliver customized educational content to participants. This research aims to explore the intricate dimensions of diabetes management through personalized data-driven approaches within the framework of the GluCare.Health hybrid model. By assessing the results of a provider that vertically integrates a care model that utilizes both virtual and physical, newer ‘hybrid’ providers can aim to elevate patient outcomes, bolster treatment adherence, and optimize the allocation of healthcare resources, all of which are not achieved optimally within traditional physical-only, or digital-only care models (17).

## 2 Methodology

### 2.1 Study design and participants

A retrospective case-control real-world observational study that involved the extraction and analysis of medical records. The study included Type 2 Diabetic patients attending the GluCare clinic, based in the UAE, Dubai, Jumeirah 1. A signed written consent was taken at the initial visit to the clinic. A total of 262 patients were included, who fit the following inclusion criteria:

- Patients previously diagnosed with Type 2 Diabetes.
- Patients who have been under the GluCare model of care for a minimum period of 3 months with a minimum of two physical visits (baseline, 3 months).

### 2.2 Exposure and outcomes

The exposure of interest in this study is the GluCare.Health Model, a hybrid diabetes management approach that integrates in-clinic care with RCDM and digital patient engagement. The outcomes of interest include improvements in glycemic control, as measured by changes in HbA1c levels, and other diabetes-related health metrics such as lipid profiles, cardiovascular (CV) risk, kidney function (eGFR), urine Albumin/Creatinine Ratio, Uric Acid, and high sensitive CRP levels, following the implementation of this model for patients with Type 2 Diabetes.

### 2.3 Classification of cohorts

The following metrics were used to classify the cases and controls. To ensure the validity of the study and minimize bias in comparing outcomes between the hybrid continuous care group (case group) and the episodic, physical-only care group (control group), careful attention was given to matching controls based on several key baseline characteristics. All patients in both the case and control groups were matched to have similar baseline characteristics, including poor glycemic control, age, sex, and weight. By controlling for these important variables, the study aimed to isolate the effect of the care model (hybrid continuous vs. episodic physical) on patient outcomes, thereby reducing the potential for confounding factors to bias the results.

#### 2.3.1 Case group

Patients were classified in this group if:

1. They had a minimum of two in-clinic visits.
2. Communication with a coach/physician/dietician/educator at least once every 2 weeks via the chat function on the app.
3. A minimum of 1 body weight reading is received on the app for every 30 days.

Case group can be classified as hybrid continuous care.

#### 2.3.2 Control group

Patients were classified as control if they had a minimum of two in-clinic visits without any virtual engagement on the app. The control group can be classified as episodic, physical only care.

### 2.4 Data collection

The extraction and collection of data was conducted from the period of September to October 2023, collection of data was for patients attending the clinic and had their 3 months follow-up from January 2021 to August 2023. The data was extracted from the physicians’ patient records (at baseline and 3 months) using the EMR (Diamond, Hicom, UK). Variables collected included patients’ gender, age, weight, height, and current diabetes-related drug intake. Laboratory variables were also extracted including HbA1c, lipid profile (total cholesterol, LDL, HDL, and triglycerides), CV risk, eGFR, urine Albumin/Creatinine Ratio, Uric Acid, and high sensitive CRP. CV risk was calculated using the United Kingdom Prospective Diabetes Study (UKPDS) risk engine algorithm in individuals with Type 2 Diabetes (13). Engagement interactions were collected from the GluCare Health Portal, interactions related to dietary, lifestyle, medications, and questions related to the continuous glucose monitoring (CGM) devices were counted.

### 2.5 Statistical analysis

Data were analyzed using SPSS software, version 29.0 (SPSS, Chicago, IL, USA). Continuous data like age, weight and laboratory values were expressed as means and standard deviations (SD) and categorical data like diabetes type, physical activity, and diabetes treatment were expressed as counts and percentages. The Paired T-test was used to compare between variables at baseline and annually and to compare pre and post-intervention outcomes. One-way ANOVA test and Chi-square test were used to correlate engagement with weight and HbA1c reductions. The P values at <0.05 were considered statistically significant.

## 3 Results

### 3.1 Basic demographics and characteristics

**Table 1** presents the basic demographics and characteristics of Type 2 diabetes patients (n=262). The mean age of the 262 patients is 50.49 ± 33.4 years. In terms of gender, 76.72% were male, while 23.28% were female.

**Table 1:** Basic Demographics and Characteristics.

Variable		Type 2 Patients (n=262)
**Age - (mean** ± **SD)**		50.49 ± 33.4
**Gender - n (%)**	**Male**	201 (76.72%)
	**Female**	61 (23.28%)

### 3.2 Baseline to 3-months changes in HbA1c based on starting HbA1c (n=262)

The data from **Table 2** underscores a clear trend where individuals in the case group with the highest initial HbA1c levels (≥9.0%) experienced the most significant HbA1c reduction of -3.67% (p < 0.001). In contrast, the control group showed a comparatively smaller and statistically non-significant reduction of -0.25% in the same high risk HbA1c category.

**Table 2:** Baseline to 3-month changes in HbA1c based on starting HbA1c in case and control cohorts.

HbA1c category	n	Baseline	3 months	Difference ± S.D	p-value
**Case group (n=162)**					
**< 7.5 %**	52	6.66 ± 0.55	6.05 ± 0.62	- 0.61 ± 0.72	<0.001*
**7.5% - 7.9%**	11	7.66 ± 0.13	6.29 ± 0.75	- 1.37 ± 0.74	<0.001*
**8.0% - 8.9%**	29	8.42 ± 0.30	6.64 ± 0.72	- 1.78 ± 0.67	<0.001*
**≥ 9.0%**	70	10.68 ± 1.34	7.01 ± 1.01	- 3.67 ± 1.62	<0.001*
**Control group (n=100)**					
**< 7.5 %**	56	6.43 ± 0.60	6.43 ± 1.00	0.00 ± 0.80	0.982
**7.5% - 7.9%**	11	7.67 ± 0.15	7.73 ± 0.89	+ 0.07 ± 0.83	0.796
**8.0% - 8.9%**	18	8.39 ± 0.29	8.03 ± 0.94	- 0.37 ± 0.92	0.108
**≥ 9.0%**	15	10.58 ± 1.64	10.33 ± 2.46	- 0.25 ± 1.89	0.616

*The P-values <0.05 indicate the statistical significance of paired sample t-test

### 3.3 Case (n=162) vs. control analysis (n=100)

Comparing Type 2 diabetes patients in the case group and control group (**Table 3**). In the case group, significant improvements were observed in various health parameters, including HbA1c (-2.19%), weight (-5.05 kg), BMI (-1.99 kg/m2), LDL (-25.3 mg/dL), total cholesterol (-31.82 mg/dL), and CVD risk (-6.50). In contrast, the control group showed smaller improvements in HbA1c (-0.10%), weight (-3.15 kg), LDL (-11.63 mg/dL) total cholesterol (-9.81 mg/dL), and CVD risk (-1.75).

**Table 3:** Cases vs. Controls analysis in Type 2 Diabetic Patients.

Variable	Case group at Baseline(n=162)	Case group at 3 months(n=162)	Mean difference ± SD	p-value	Control group at Baseline(n=100)	Control group at 3 months (n=100)	Mean difference ± SD	p-value
**HbA1c (%)**	8.78 ± 2.0	6.58 ± 0.92	- 2.19 ± 1.79	<0.001*	7.53 ± 1.6	7.44 ± 1.8	- 0.10 **±** 1.05	0.368
**Weight (kg)**	87.84 ± 18.9	82.79 ± 18.1	- 5.05 ± 6.52	<0.001*	83.37 ± 17.4	80.22 ± 18.5	- 3.15 ± **0**.17	<0.001*
**BMI (kg/m2)**	31.28 ± 9.1	29.29 ± 7.6	- 1.99 ± 4.72	<0.001*	30.27 ± 9.5	28.27 ± 4.8	- 1.86 **±** 8.54	0.033*
**LDL (mg/dL)**	120.9 ± 40.3	95.60 ± 41.7	- 25.3 ± 42.4	<0.001*	112.99 ± 42.0	101.36 ±38.1	- 11.63 **±** 42.04	0.008*
**CVD risk**	15.83 ± 17.4	9.33 ± 12.8	- 6.50 ± 12.54	<0.001*	14.2 ± 14.3	12.45 ± 12.7	- 1.75 **±** 8.18	0.042*
**eGFR**	112.56 ± 31.5	109.92 ± 30.4	- 2.64 ± 15.34	0.032*	109.02 ± 28.8	104.06 ± 33.5	- 4.96 **±** 23.81	0.046*
**Cholesterol (mg/dL)**	186.34 ± 48.0	154.53 ± 44.6	- 31.82 ± 50.98	<0.001*	172.11 ± 50.4	162.3 ± 48.2	- 9.81 **±** 43.85	0.032*
**Urine Albumin/Creatinine Ratio**	71.52 ± 285.8	44.36 ± 224.23	- 27.16 ± 117.27	0.007*	143.28 ± 501.6	101.43 ± 371.3	- 41.86 ± 259.46	0.143
**HDL (mg/dL)**	43.62 ± 11.6	43.94 ± 11.9	+0.32 ± 6.15	0.513	46.30 ± 10.4	46.16 ± 9.9	- 0.14 ± 6.77	0.842
**Triglycerides (mg/dL)**	249.47 ± 291.2	222.37 ± 886.9	- 27.1 ± 715.6	0.638	195.07 ± 136.8	206.54 ± 189.4	+11.47 **±** 157.97	0.483
**Uric Acid**	6.14 ± 9.1	6.02 ± 10.4	- 0.12 ± 3.60	0.669	5.39 ± 1.3	5.21 ± 1.3	- 0.18 ± 1.10	0.115
**CRP**	1.34 ± 5.8	1.13 ± 5.3	- 0.21 ± 1.46	0.081	1.12 ± 4.5	1.28 ± 5.2	+ 0.17 **±** 2.70	0.557

*The P-values <0.05 indicate the statistical significance of paired sample t-test

### 3.4 Correlation analysis of virtual interactions and clinical outcomes - case analysis

**Figure 1:**
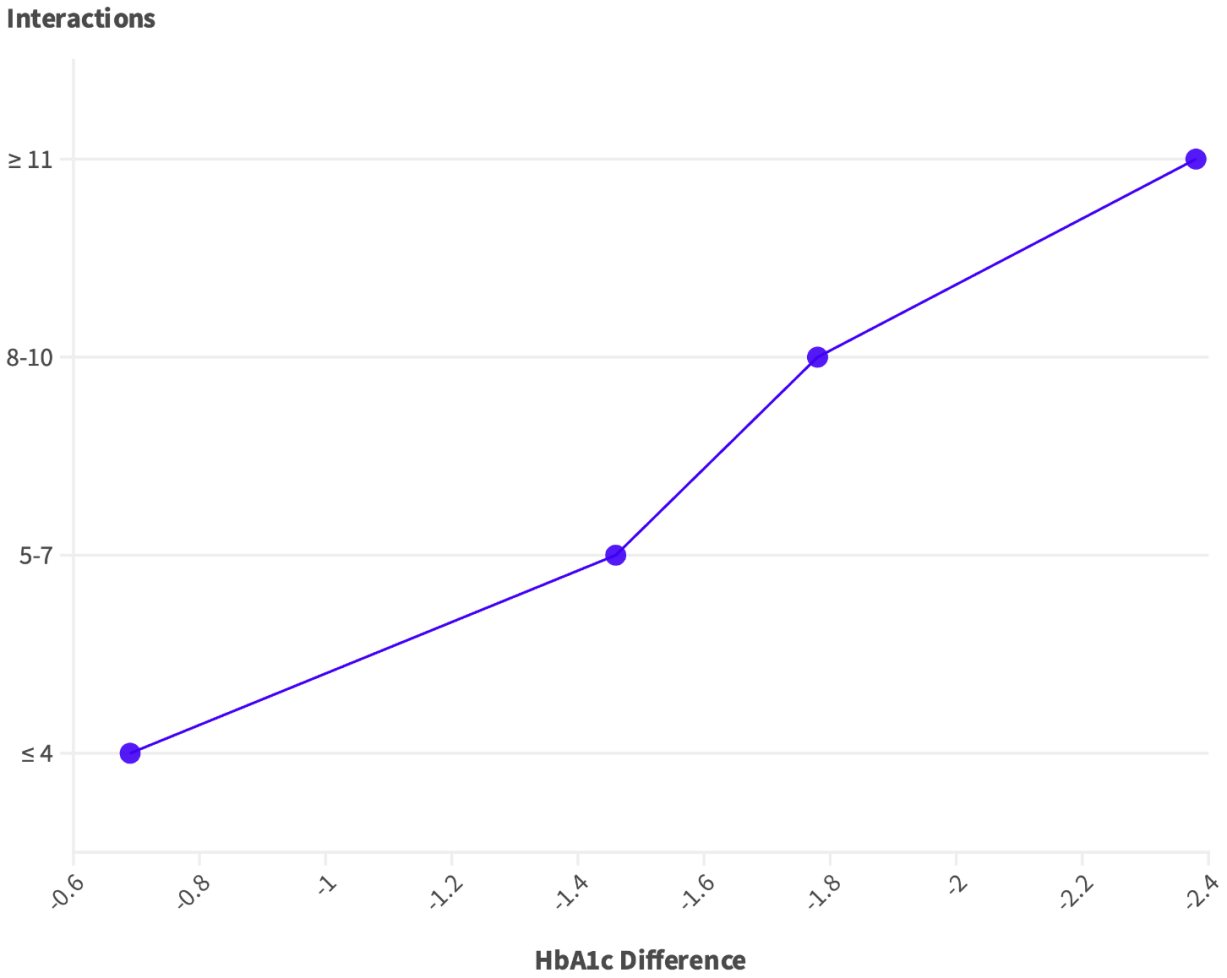
Trends of HbA1c difference as per engagement groups.

**Figure 2:**
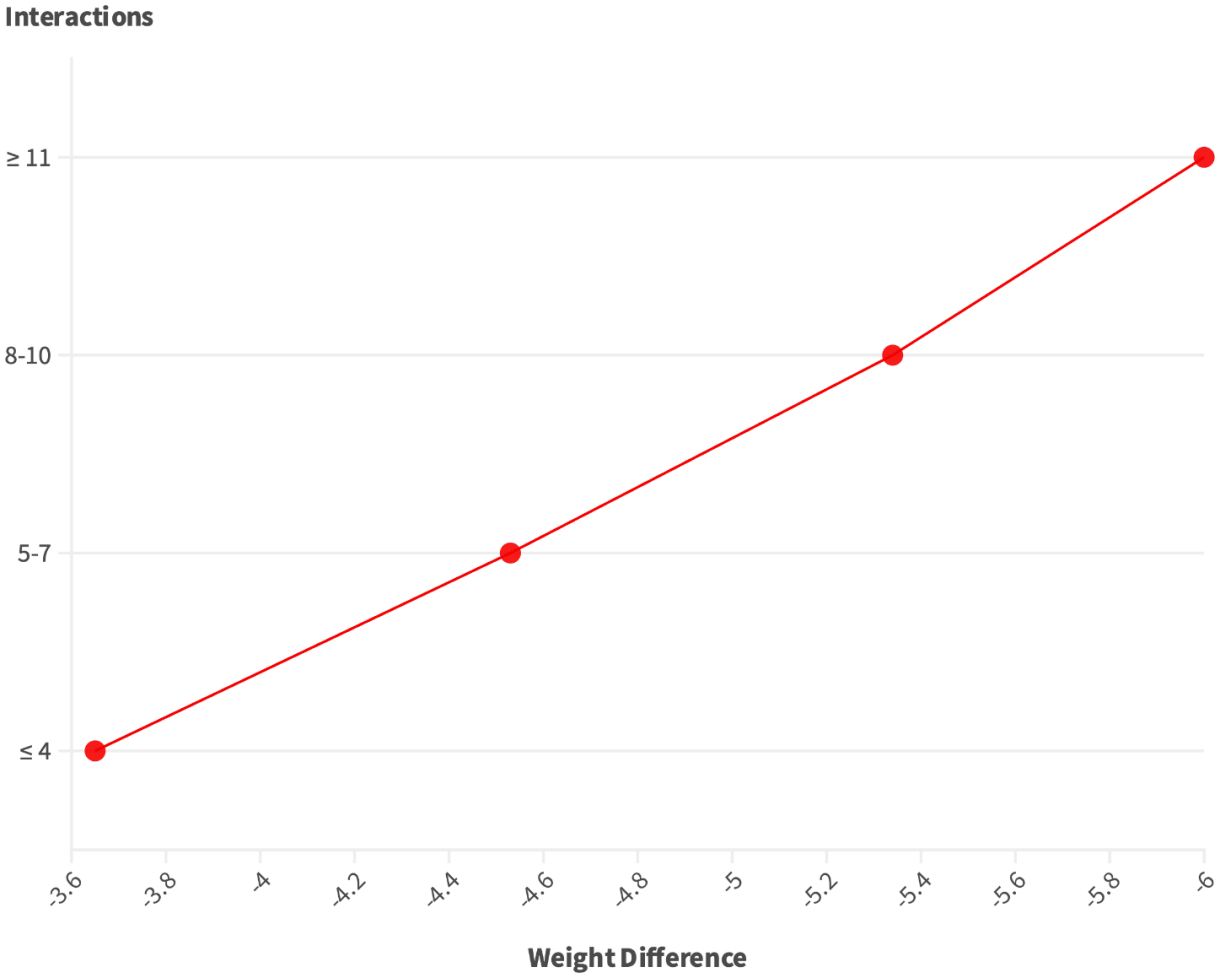
Trends of HbA1c difference as per engagement groups.

In **Table 4**, there is a correlation between engagement interactions and clinical outcomes for the case group (n=162). The table illustrates the mean changes in HbA1c, and weight reduction based on different levels of engagement interactions. The engagement interactions showed a mean of 15.28 ± 20.1 for inbound interactions and 25.93 ± 20.1 for outbound interactions, averaging a total of 20.8 interactions over 90 days. Notably, as the number of interactions increases, there is a trend of greater improvements in HbA1c and weight reduction. Specifically, for individuals with ≥11 interactions over the course of 90 days, there was a statistically significant reduction in HbA1c (p= 0.027) and weight (p = 0.004) with reductions of -2.38 and -6.00 kg being achieved, **Figures 1** and **2**. Illustrates the trend in improvement for engagement levels.

**Table 4:** Correlation between Engagement Interactions with Clinical Outcomes (n=119).

	Category			Mean ± SD		
**Engagement Interactions**	**Inbound**			15.28 ± 20.1		
	**Outbound**			25.93 ± 20.1		
	**Total**			20.80 ± 20.98		
	**Virtual Interactions**					
**Outcome**	**≤ 4 interactions (n=34)**	**5- 7 interactions (n=14)**	**8-10 interactions (n=13)**		**≥ 11 interactions (n=58)**	**P-value**
**HbA1c Reduction**	- 0.69 ± 1.8	- 1.46 ± 1.21	-1.78 ± 1.67		-2.38 ± 1.9	0.027*
**Weight Reduction**	-3.65 ± 3.0	-4.53 ± 4.2	- 5.34 ± 4.5		-6.00 ± 9.4	0.004*

*The P-values <0.05 indicate the statistical significance of one-way ANOVA test

## 4 Discussion

The present study highlights the results of a hybrid care model, integrating in-clinic and remote monitoring components, with promising 90 days outcomes in the management of Type 2 diabetes. On average, patients within the case group achieved an HbA1c of < 7. The amalgamation of traditional healthcare delivery with modern technology aims to address the multifaceted challenges posed by diabetes and enhance patient outcomes. Our findings highlight significant improvements in various clinical parameters, emphasizing the potential of the hybrid models as an effective and comprehensive approach to diabetes care.

Comparing the GluCare Health model with digital-only approaches underscores the importance of a hybrid model. Studies have reported that, the overall effect of mobile phone interventions only without physical interactions, have resulted in a mean -0.5% (95% CI 0.3% - 0.7%) reduction in HbA1c over a 6-month follow-up (18). A meta-analysis, measuring the effect of telemedicine on improvements in HbA1c reductions, reported the reductions in HbA1c by -0.57%, -0.28%, -0.26% at ≤ 3 months, 4–12 months and > 12 months, respectively (19). The present study reported 3 months of improvements in HbA1c by -2.19% in the case group with improvements highly correlated to overall engagement numbers. The control group showed no significant improvement in major diabetes parameters, mimicking the ineffectiveness seen in physical-only care models. Similar results were seen over a long management period (12 months) using the GluCare hybrid model demonstrating a reduction in HbA1c by -1.54% with overall HbA1c <7.0% as recommended by the ADA (10, 20). The present study also reported improvements in HbA1c as categorized by different baseline levels. Those who were furthest from their HbA1c goals at the start of the program (baseline HbA1c ≥ 9.0%) achieved the greatest improvement in HbA1c, with an average change of - 3.67% in the case group and by only -0.25% in the control group. Compared to a Digitally Enhanced Diabetes Self-Management Education and Support (DSMES) Program (no physical infrastructure) which showed HbA1c reductions of -1.4% in patients with poorly controlled diabetes (HbA1c ≥9.0%) (21), the hybrid model is nearly two and a half times more effective. Reduction in HbA1c in diabetic patients has proven positive outcomes with each 1.0% decrease in HbA1c linked to a 21% risk reduction in any diabetes-related end point, a 22% risk reduction in diabetes-related death, and a 14% risk reduction in all-cause mortality (p = 0.0001) (22). Moreover, a recent study showed from multivariable analyses that, for every 1% decrease in HbA1c it was associated with a 2% decrease in all-cause total healthcare costs and a 13% decrease in diabetes-related total healthcare costs (23).

In addition to controlled glycemia profile, significant improvements in lipid profile, kidney functions, and reduced CV risk were observed in the present study. We found a consistent impact on cardiovascular outcomes among the case group with patients exhibiting a significant reduction in weight, BMI, LDL, cholesterol, eGFR, and urine albumin/calcium ratio. Given that all these patients were already under medication and receiving care from an existing healthcare provider, it highlights the constraints of traditional care systems primarily centered on medication therapy. Case group patients with poor baseline control managed to lower their HbA1c to 6.58%, falling below the recommended ADA guidelines (20). Interventions likely played a pivotal role in promoting healthier habits, medication adherence, and overall diabetes management. Several of these studies measuring the impact of digital health have omitted reporting on other clinical parameters or medication alterations, mainly because these programs were not integrated into the primary care providers’ management strategies (21, 24, 25). Instead, they serve as supplementary management and engagement tools, typically led by health coaches and educators to assist patients with no direct engagement with the primary caregiver team. The incorporation of a hybrid care model into a diabetes management solution could potentially enhance HbA1c control (26), possibly attributed to a more comprehensive end-to-end approach and increased engagement, as demonstrated in this study. Enabling a diversified care team to access both remote and EMR data facilitates, utilizing more data-driven behavioral nudges or personalized engagement can potentially result in more targeted interventions for effective diabetes management (10).

The platform’s engagement was strong as evidenced by the high frequency of use across the features of the digital platform. Overall, the patients had an average of 15.28 interactions during the 3 months. This level of interaction is indicative of the positive impact of digital solutions on clinical parameters. These interactions not only enhance patient understanding but also provide a valuable avenue for addressing concerns, seeking clarification, and receiving personalized guidance. The correlational analysis between the number of interactions and the change in HbA1c and weight was clearly visible. Patients with a high number of engagements (≥ 11) had significant reductions in HbA1c by -2.38% and weight by -6.00 kg. Similarly, in a study reporting the improvement of web-based management compared to usual care, regular data uploads were more likely to achieve and maintain reductions in patients’ HbA1c by -1.0% (13). However, the results of the present study were superior to digital-only interventions.

The study demonstrates several strengths, including its real-world setting, the comprehensive nature of the data collected, and the use of a matched case-control design which enhances the validity of the findings by reducing potential biases related to patient characteristics. The integration of a hybrid care model, combining both in-person and digital interventions, provides a novel approach to diabetes management, addressing both the physical and behavioral aspects of the disease. Furthermore, the study’s focus on a wide range of clinical outcomes, beyond just glycemic control, allows for a more holistic understanding of the impact of the GluCare.Health Model on overall patient health. The utilization of continuous data collection through digital means offers an innovative method of patient engagement and monitoring, potentially leading to more timely and personalized interventions. However, the study is not without limitations. The retrospective, observational design, while valuable for examining real-world outcomes, does not allow for causal inferences to be made with the same level of certainty as randomized controlled trials.

## 5 Conclusion

In conclusion, the GluCare Health hybrid model, as highlighted in this study, presents a promising and new comprehensive approach to the management of Type 2 diabetes. The integration of in-clinic and remote monitoring components has demonstrated significant improvements in various clinical parameters, particularly a remarkable reduction in HbA1c levels (-2.19%) regardless of baseline within the first 90 days. All patients following the hybrid approach had HbA1c < 7 within 90 days in comparison to patients who followed a traditional care approach where the average HbA1c was 7.4%. The study’s findings not only showcase controlled glycemia profiles but also reveal substantial enhancements in LDL (-25.3 mg/dL), total cholesterol (-31.82 mg/dL), and reduced cardiovascular risk (-6.50) among the case group. The platform’s strong engagement, with an average of 15.28 interactions per patient over three months, indicates the positive impact of hybrid solutions on clinical parameters with a direct correlation between outcomes and engagement. Those who had engagement >11 over 90 days, regardless of engagement type, had the highest HbA1c and weight reduction. These results suggest that the GluCare hybrid model’s end-to-end approach, combining physical locations and remote digital solutions, has the potential to significantly improve diabetes management outcomes through increased patient engagement and a more data-driven, targeted intervention strategy.

## Data availability statement

The original contributions presented in the study are included in the article/**Supplementary Material**. Further inquiries can be directed to the corresponding author.

## Ethics statement

This study was conducted in strict adherence to the ethical standards of the Declaration of Helsinki. Informed consent was obtained from all individual participants included in the study. Clinical practices were regulated under the healthcare authority of the United Arab Emirates, which ensures compliance with international ethical standards for research involving human subjects. 

## Author contributions

HZ: Conceptualization, Data curation, Formal analysis, Investigation, Methodology, Supervision, Validation, Writing – original draft. YS: Conceptualization, Writing – review & editing. SA: Data curation, Writing – review & editing. JB: Data curation, Writing – review & editing. MD: Data curation, Writing – review & editing. JD: Data curation, Writing – review & editing. ND: Data curation, Writing – review & editing. DD: Data curation, Writing – review & editing. AH: Conceptualization, Writing – review & editing. IA: Conceptualization, Investigation, Writing – original draft.
