# Supplementary material for: Measuring the effectiveness of hybrid diabetes care over 90 days through continuous data monitoring in type 2 diabetic patients
Source: Front Endocrinol (Lausanne). 2024 May 7;15:1355792. doi: 10.3389/fendo.2024.1355792 (PMC11106412; doi:10.3389/fendo.2024.1355792)
Supplement: Supplementary file 1 [file DataSheet_1.docx]

Supplementary Material

# Supplementary Figures and Tables

## Supplementary Tables

Table 1: Basic Demographics and Characteristics

| **Variable** |  | **Type 2 Patients (n=262)** |
| --- | --- | --- |
| **Age - (mean** ± **SD)** |  | 50.49 ± 33.4 |
| **Gender - n (%)** | **Male** | 201 (76.72%) |
|  | **Female** | 61 (23.28%) |

Table 2: Baseline to 3-month changes in HbA1c based on starting HbA1c in case and control cohorts

| **HbA1c category** | **n** | **Baseline** | **3 months** | **Difference ± S.D** | **p-value** |
| --- | --- | --- | --- | --- | --- |
| **Case group (n=162)** | | | | | |
| **< 7.5 %** | 52 | 6.66 ± 0.55 | 6.05 ± 0.62 | - 0.61 ± 0.72 | <0.001* |
| **7.5% - 7.9%** | 11 | 7.66 ± 0.13 | 6.29 ± 0.75 | - 1.37 ± 0.74 | <0.001* |
| **8.0% - 8.9%** | 29 | 8.42 ± 0.30 | 6.64 ± 0.72 | - 1.78 ± 0.67 | <0.001* |
| **≥ 9.0%** | 70 | 10.68 ± 1.34 | 7.01 ± 1.01 | - 3.67 ± 1.62 | <0.001* |
| **Control group (n=100)** | | | | | |
| **< 7.5 %** | 56 | 6.43 ± 0.60 | 6.43 ± 1.00 | 0.00 ± 0.80 | 0.982 |
| **7.5% - 7.9%** | 11 | 7.67 ± 0.15 | 7.73 ± 0.89 | + 0.07 ± 0.83 | 0.796 |
| **8.0% - 8.9%** | 18 | 8.39 ± 0.29 | 8.03 ± 0.94 | - 0.37 ± 0.92 | 0.108 |
| **≥ 9.0%** | 15 | 10.58 ± 1.64 | 10.33 ± 2.46 | - 0.25 ± 1.89 | 0.616 |

*****The P-values <0.05 indicate the statistical significance of paired sample t-test

Table 3: Cases vs. Controls analysis in Type 2 Diabetic Patients

| **Variable** | **Case group at Baseline**  **(n=162)** | **Case group at 3 months**  **(n=162)** | **Mean difference ± SD** | **p-value** | **Control group at Baseline**  **(n=100)** | **Control group at 3 months (n=100)** | **Mean difference ± SD** | **p-value** |
| --- | --- | --- | --- | --- | --- | --- | --- | --- |
| **HbA1c (%)** | 8.78 ± 2.0 | 6.58 ± 0.92 | - 2.19 ± 1.79 | <0.001* | 7.53 ± 1.6 | 7.44 ± 1.8 | - 0.10 **±** 1.05 | 0.368 |
| **Weight (kg)** | 87.84 ± 18.9 | 82.79 ± 18.1 | - 5.05 ± 6.52 | <0.001* | 83.37 ± 17.4 | 80.22 ± 18.5 | - 3.15 ± **0**.17 | <0.001* |
| **BMI (kg/m2)** | 31.28 ± 9.1 | 29.29 ± 7.6 | - 1.99 ± 4.72 | <0.001* | 30.27 ± 9.5 | 28.27 ± 4.8 | - 1.86 **±** 8.54 | 0.033* |
| **LDL (mg/dL)** | 120.9 ± 40.3 | 95.60 ± 41.7 | - 25.3 ± 42.4 | <0.001* | 112.99 ± 42.0 | 101.36 ±38.1 | - 11.63 **±** 42.04 | 0.008* |
| **CVD risk** | 15.83 ± 17.4 | 9.33 ± 12.8 | - 6.50 ± 12.54 | <0.001* | 14.2 ± 14.3 | 12.45 ± 12.7 | - 1.75 **±** 8.18 | 0.042* |
| **eGFR** | 112.56 ± 31.5 | 109.92 ± 30.4 | - 2.64 ± 15.34 | 0.032* | 109.02 ± 28.8 | 104.06 ± 33.5 | - 4.96 **±** 23.81 | 0.046* |
| **Cholesterol (mg/dL)** | 186.34 ± 48.0 | 154.53 ± 44.6 | - 31.82 ± 50.98 | <0.001* | 172.11 ± 50.4 | 162.3 ± 48.2 | - 9.81 **±** 43.85 | 0.032* |
| **Urine Albumin/Creatinine Ratio** | 71.52 ± 285.8 | 44.36 ± 224.23 | - 27.16 ± 117.27 | 0.007* | 143.28 ± 501.6 | 101.43 ± 371.3 | - 41.86 ± 259.46 | 0.143 |
| **HDL (mg/dL)** | 43.62 ± 11.6 | 43.94 ± 11.9 | +0.32 ± 6.15 | 0.513 | 46.30 ± 10.4 | 46.16 ± 9.9 | - 0.14 ± 6.77 | 0.842 |
| **Triglycerides (mg/dL)** | 249.47 ± 291.2 | 222.37 ± 886.9 | - 27.1 ± 715.6 | 0.638 | 195.07 ± 136.8 | 206.54 ± 189.4 | +11.47 **±** 157.97 | 0.483 |
| **Uric Acid** | 6.14 ± 9.1 | 6.02 ± 10.4 | - 0.12 ± 3.60 | 0.669 | 5.39 ± 1.3 | 5.21 ± 1.3 | - 0.18 ± 1.10 | 0.115 |
| **CRP** | 1.34 ± 5.8 | 1.13 ± 5.3 | - 0.21 ± 1.46 | 0.081 | 1.12 ± 4.5 | 1.28 ± 5.2 | + 0.17 **±** 2.70 | 0.557 |

*****The P-values <0.05 indicate the statistical significance of paired sample t-test

Table 4: Correlation between Engagement Interactions with Clinical Outcomes (n=119)

|  | **Category** | | | **Mean ± SD** | | |
| --- | --- | --- | --- | --- | --- | --- |
| **Engagement Interactions** | **Inbound** | | | 15.28 ± 20.1 | | |
|  | **Outbound** | | | 25.93 ± 20.1 | | |
|  | **Total** | | | 20.80 ± 20.98 | | |
| **Outcome** | **Virtual Interactions** | | | | | |
|  | **≤ 4 interactions (n=34)** | **5- 7 interactions (n=14)** | **8-10 interactions (n=13)** | | **≥ 11 interactions (n=58)** | **P-value** |
| **HbA1c Reduction** | - 0.69 ± 1.8 | - 1.46 ± 1.21 | -1.78 ± 1.67 | | -2.38 ± 1.9 | 0.027* |
| **Weight Reduction** | -3.65 ± 3.0 | -4.53 ± 4.2 | - 5.34 ± 4.5 | | -6.00 ± 9.4 | 0.004* |

*****The P-values <0.05 indicate the statistical significance of one-way ANOVA test

## Supplementary Figures


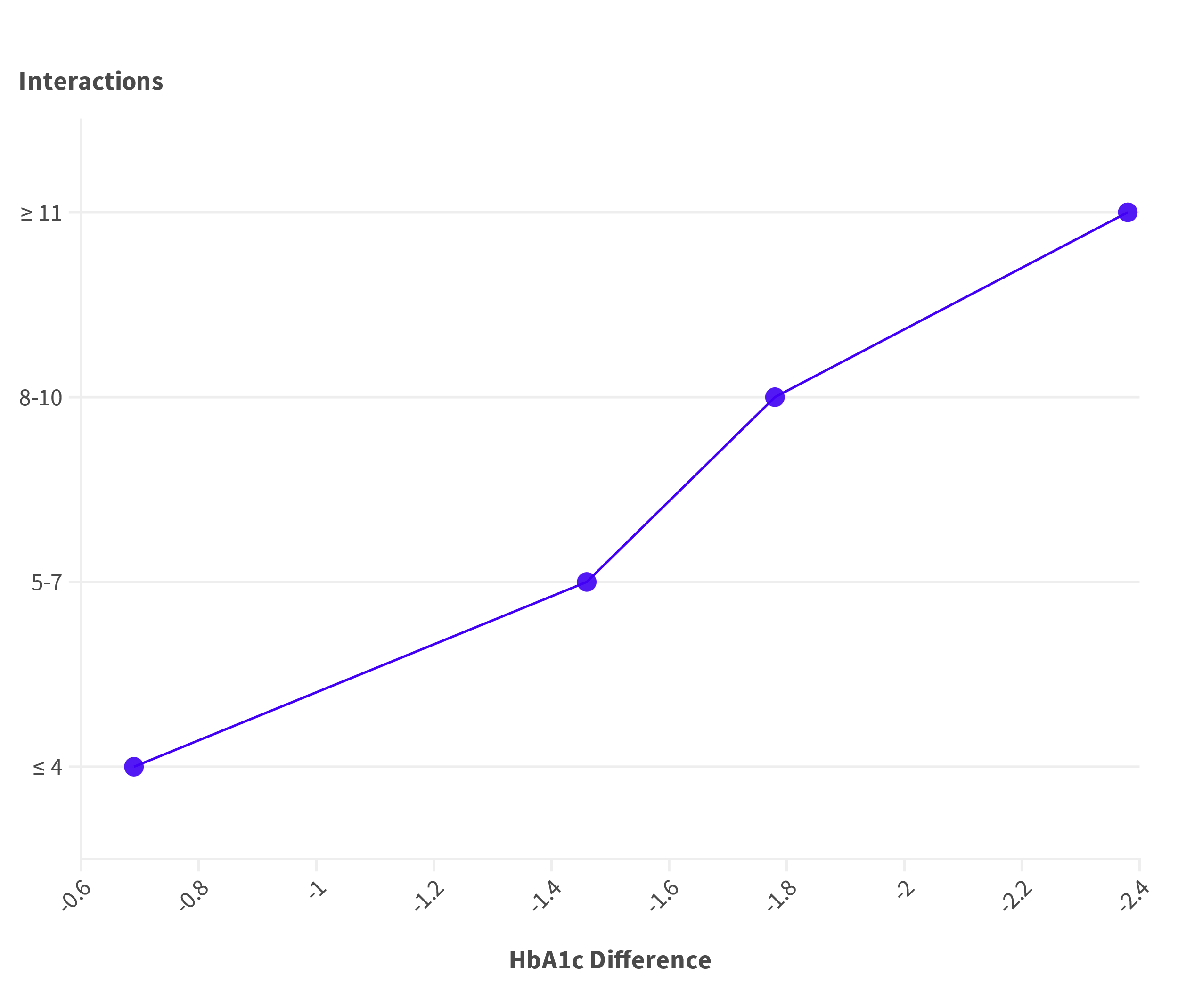


Figure 1: Trends of HbA1c difference as per engagement groups.


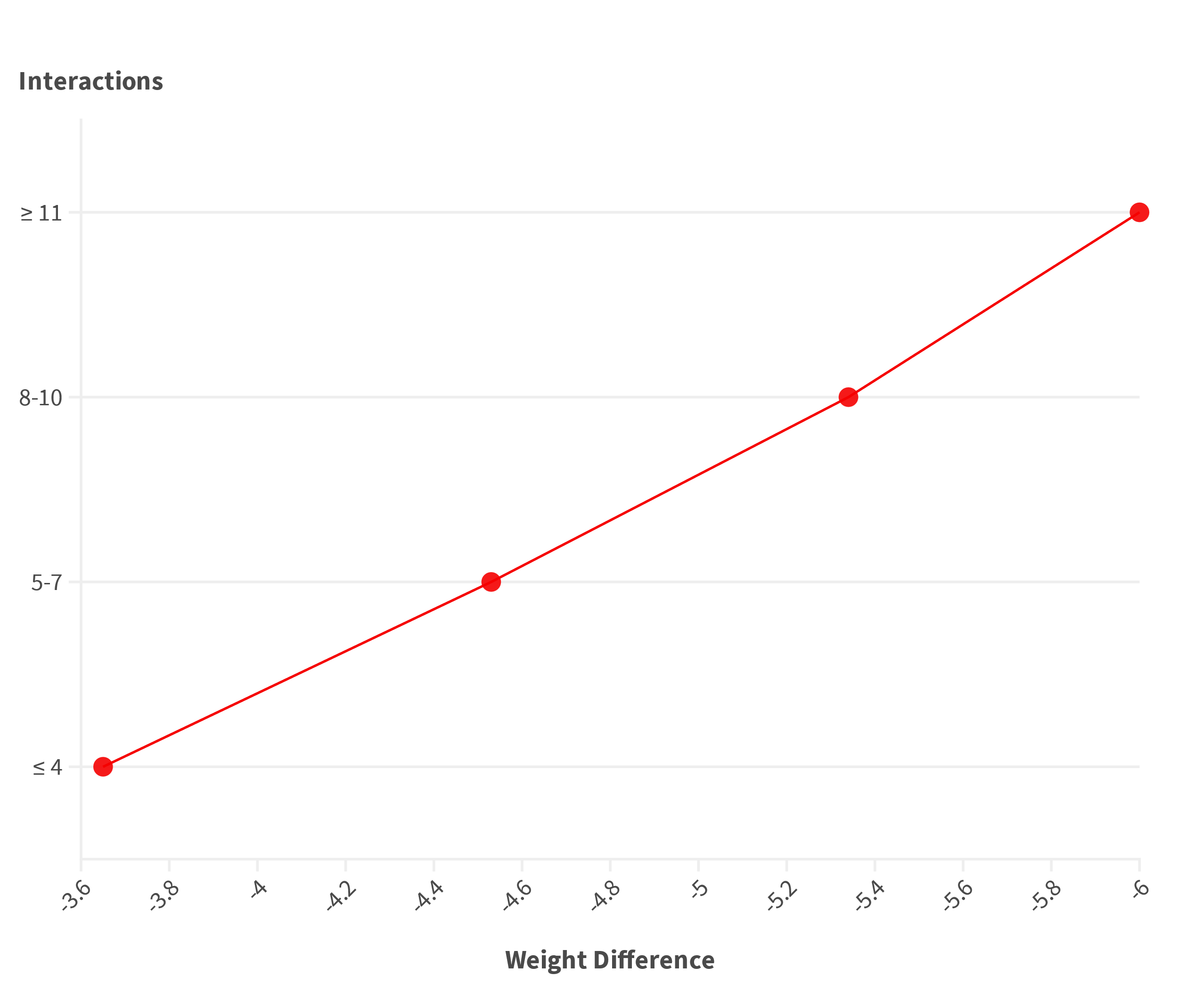


Figure 2: Trends of weight difference as per engagement groups.
